# miR-19b-3p is associated with a diametric response to resistance exercise in older adults and regulates skeletal muscle anabolism via PTEN inhibition

**DOI:** 10.1152/ajpcell.00190.2021

**Published:** 2021-10-27

**Authors:** Donato A. Rivas, Fei Peng, Townsend Benard, Adelino Sanchez Ramos da Silva, Roger A. Fielding, Lee M. Margolis

**Affiliations:** ^1^Nutrition, Exercise Physiology and Sarcopenia Laboratory, Jean Mayer USDA Human Nutrition Research Center on Aging at Tufts University, Boston, Massachusetts; ^2^School of Physical Education and Sport of Ribeirão Preto, University of São Paulo, Ribeirão Preto, Brazil; ^3^Military Nutrition Division, US Army Research Institute of Environmental Medicine, Natick, Massachusetts

**Keywords:** aging, exercise, microRNA, sarcopenia, skeletal muscle

## Abstract

Understanding paradoxical responses to anabolic stimulation and identifying the mechanisms for this inconsistency in mobility-limited older adults may provide new targets for the treatment of sarcopenia. Our laboratory has discovered that dysregulation in microRNA (miRNA) that target anabolic pathways is a potential mechanism resulting in age-associated decreases in skeletal muscle mass and function (sarcopenia). The objective of the current study was to assess circulating miRNA expression profiles in diametric response of leg lean mass in mobility-limited older individuals after a 6-mo progressive resistance exercise training intervention (PRET) and determine the influence of differentially expressing miRNA on regulation of skeletal muscle mass. Participants were dichotomized by gain (Gainers; mean +561.4 g, *n* = 33) or loss (Losers; mean −589.8 g, *n* = 40) of leg lean mass after PRET. Gainers significantly increased fat-free mass 2.4% vs. −0.4% for Losers. Six miRNA (miR-1-3p, miR-19b-3p, miR-92a, miR-126, miR-133a-3p, and miR-133b) were significantly identified to be differentially expressed between Gainers and Losers, with miR-19b-3p being the miRNA most highly associated with increases in fat-free mass. Using an aging mouse model, we then assessed if miR-19b-3p expression was different in young mice with larger muscle mass compared with older mice. Circulating and skeletal muscle miR-19b-3p expression was higher in young compared with old mice and was positively associated with muscle mass and grip strength. We then used a novel integrative approach to determine if differences in circulating miR-19b-3p potentially translate to augmented anabolic response in human skeletal muscle cells in vitro. Results from this analysis identified that overexpression of miR-19b-3p targeted and downregulated PTEN by 64% to facilitate significant ∼50% increase in muscle protein synthetic rate as measured with SUnSET. The combine results of these three models identify miR-19b-3p as a potent regulator of muscle anabolism that may contribute to an inter-individual response to PRET in mobility-limited older adults.

## INTRODUCTION

Age-associated decreases in skeletal muscle mass and function (sarcopenia) limit older individuals’ mobility and independence and are risk factors for mortality (1). Changes in skeletal muscle size are fundamentally determined by dynamic interactions between rates of muscle protein synthesis and protein breakdown (2–6). Ultimately, increasing muscle size requires a chronic net positive protein balance, favoring muscle protein synthesis over protein breakdown (5). Molecular processes regulating muscle protein turnover are complex cellular pathways that include alterations to signaling proteins, gene transcription, and microRNA (miRNA) expression (7, 8). In particular, there is abundant evidence showing that exercise and nutrition are powerful independent stimulators of skeletal muscle protein synthesis with the ability to shift net protein balance from negative to positive (9–11). Therefore, a combination of resistance exercise training and nutrients could augment the individual effects of both these stimuli on anabolic signaling, transcriptional pathways, and posttranscriptional regulators.

It is widely accepted that strategies such as adequate protein intake (∼1.2 g/kg/day) and physical training (resistance exercise) are the critical interventions that can help maintain or increase skeletal muscle mass during aging (12, 13). However, elderly adults show a blunted muscle protein synthetic response (“anabolic resistance”) to amino acid administration and physical activity when compared with young adults (14–17). Impairments in protein digestion and amino acid absorption, insulin-mediated muscle tissue perfusion, amino acid uptake in muscle, or reduced activation of key signaling proteins and transcriptional regulators have all been proposed as potential contributors to anabolic resistance (17–22). In addition, a number of studies have reported that the blunted anabolic response in healthy older adults compared with younger adults may be due to divergent expressions of miRNA that govern molecular pathways that regulate muscle mass (21, 23–25). Our previous work utilized miRNA expression profiles in circulation (25) and skeletal muscle (21) from young and older individuals before and after an acute bout of high-intensity resistance exercise. Specifically, we determined relationships for individual miRNA and miRNA families with muscle plasticity, metabolic health and as predictive of age, and age-associated changes in body composition.

An additional complication that may negatively influence muscle growth with aging is the inter-individual variability or heterogeneity in exercise adaptation responses (26–31). Some individuals may exhibit an inadequate response to exercise or nutrition interventions relative to age-matched peers, resulting in a lower hypertrophic response. Understanding the variations or paradoxical responses to anabolic stimulation and identifying the mechanisms for this inconsistency in mobility-limited older adults may provide new targets for sarcopenia treatment.

In the current study, we found a diametric response of leg lean mass in mobility-limited older individuals after a 6-mo progressive resistance exercise training intervention (PRET). Participants were dichotomized by gain (Gainers) or loss (Losers) of leg lean mass after PRET. The Losers experienced declines in body mass due to loss of fat mass. In contrast, Gainers increased body mass due to a significant increase in fat-free mass. Therefore, we used a novel integrative, translational approach to determine if extracellular miRNAs previously uncovered by unbiased analysis contribute to the anabolic heterogeneity to exercise training in mobility-limited older adults and if the addition of a whey protein isolate during exercise can influence miRNA expression. Six miRNA (miR-1-3p, miR-19b-3p, miR-92a, miR-126, miR-133a-3p, and miR-133b) were identified to be differentially expressed between Gainers and Losers, with miR-19b-3p being the miRNA most highly associated with increases in fat-free mass. Mechanistically, we determined if manipulating the expression of miR-19b-3p, a key member of the miR-17–92 cluster, in vitro could augment anabolic response in human skeletal muscle cells. Lastly, we determined if age-related muscle atrophy and weakness are associated with miR-19b-3p expression in the skeletal muscle of a preclinical model of aging mice.

## METHODS AND MATERIALS

### Participants

A total of 80 community-dwelling older (70–85 yr old) men and women participated in the main study (32). Of the 80 individuals who participated in the parent study, data on body composition changes were available on 73 participants. Informed consent was obtained from each participant before data collection. Tufts University Health Sciences Campus Institutional Review Board approved this study (IRB No.: 8415). This investigation was registered at ClinicalTrials.gov (Trial registration: Effect of Whey Protein Supplementation and Resistance Exercise on Muscle Parameters in Older Adults, https://clinicaltrials.gov/ct2/show/NCT00635739). Data presented in the current manuscript were exploratory analysis secondary to the primary aim of the parent study. All participants were weight stable (loss or gain of body mass was less than 7.5% within the previous 6 mo), normal to slightly obese (body mass index: 21–32.5), not participating in structured exercise 6 mo before starting the study, and were functionally limited [short physical performance battery (SPPB) score ≤ 10].

### Resistance Training Protocol

The resistance training protocol was a supervised progressive program, three times per week for 6 mo, which entailed leg press, seated row, leg extension, chest press, and leg curl. Participants performed the training exercises progressing to 80% of their one-repetition maximum (1-RM). The 1-RM was assessed at baseline and reevaluated monthly. Participants initially performed 2 sets of 10 repetitions progressing to 3 sets of 12 repetitions throughout the 6-mo intervention period. Each set was followed by a 1- to 2-min rest period. Resistance training was preceded by 5 min of lower extremity “warm-up” (walking or stationary cycling). All training was performed on Cybex VR2 machines (Cybex International, Medway, MA). Adherence to resistance training was quantified by average attendance at scheduled sessions and the average exercise intensity (% 1-RM) for the knee extension and leg press exercises achieved over the length of the trial.

### Participant Dichotomization

**Figure 1.**
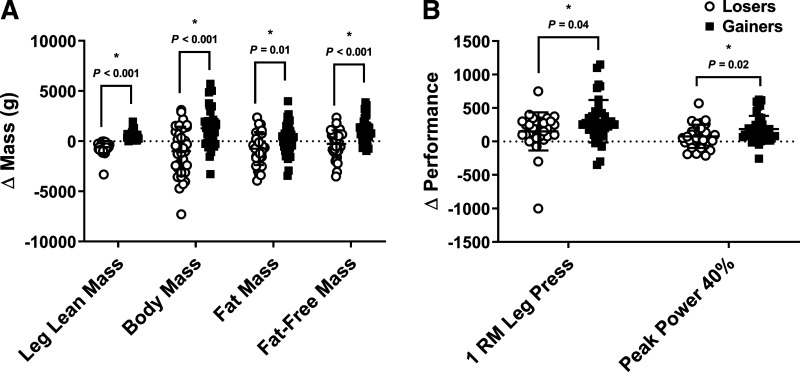
Change in leg lean mass, whole body mass, fat mass and lean mass by dual energy X-ray absorptiometry (*A*), and lower limb 1-repetition maximum, peak power 40% and peak power 70% (*B*). Data represent means ± SD (Losers; *n* = 33 and Gainers; *n* = 40). Dots are individual data points. *Different than Losers; *P* < 0.05. Statistical analysis unpaired *t* test.

Participants in this investigation were part of a more extensive randomized, double-blind, controlled study that assessed the influence of twice daily consumption of either a whey protein supplement (20-g protein, 25-g maltodextrin, 1-g fat, 189 kcal) or isocaloric control (45 g maltodextrin, 1 g fat, 189 kcal) beverage (Innovative Food Processors Inc., Faribault, MN) on change in body composition and physical function following 6 mo of resistance exercise training (32). The primary outcome of the parent investigation was that resistance exercise training independent of protein supplementation resulted in increased fat-free mass and improved lower limb strength and power, stair-climb time, chair rise, and short physical performance battery (SPPB) score (32). However, the individual data assessment indicated a large variance in response to the 6-mo resistance exercise protocol, independent of dietary treatment. As such, body composition data were assessed to identify a dichotomization variable. Change in leg lean mass was identified as a variable that dichotomized participants in those who lost (Losers; *n* = 33; −590 ± 105 g) or gained (Gainers; *n* = 40; 561 ± 62 g) mass, with no individual having a net zero change (Fig. 1*A*). There were no differences in baseline age, SPPB score, number of males and females or participants receiving whey protein supplement versus isocaloric control between Losers and Gainers (Table 1). Energy and macronutrient intake was not different between Losers and Gainers (Table 2).

**Table 1.** Group distribution and body composition^1^

	Losers	Gainers
Age, yr	78 ± 6	77 ± 6
Females, *n*	19	24
Males, *n*	14	16
Whey, *n*	18	21
Placebo, *n*	15	19
^2^SPPB score	8.5 ± 1.1	8.5 ± 1.3

Values means ± SD. No differences in baseline characteristics between losers and gainers. ^2^Short physical performance battery. Statistical analysis unpaired *t* test.

**Table 2.** Dietary intake^1^

	Losers	Gainers
Absolute		
Energy, Kcal	1,804 ± 448	1,719 ± 424
Protein, g	80 ± 23	79 ± 25
Carbohydrate, g	245 ± 63	216 ± 63
Fat, g	58 ± 29	58 ± 19
Relative		
Energy, Kcal·kg^−1^	24.07 ± 6.26	23.26 ± 5.63
Protein, g·kg^−1^	1.08 ± 0.34	1.07 ± 0.32
Carbohydrate, g·kg^−1^	3.28 ± 0.92	2.92 ± 0.95
Fat, g·kg^−1^	0.76 ± 0.40	0.79 ± 0.25

Values/means ± SD. ^1^Dietary intake determined using 3-day food records. Food records were reviewed by research dietitian for completeness. Dietary intake data were analyzed using nutrition data system for research software version 2007 (nutrition coordinating center, University of Minnesota, Minneapolis, MN). No differences between losers and gainers. Statistical analysis unpaired *t* test.

### Body Composition

Whole body and regional fat and fat-free mass were assessed using dual X-ray absorptiometry (DXA, Hologic Discovery A v12.3, Hologic Inc., Bedford, MA). Cross-sectional area (CSA) was measured by computed tomography (CT) on the nondominant thigh at the midpoint of the femur using a Siemens Somotom Scanner (Erlangen, Germany). Scans were analyzed for normal-density muscle [35–100 Houndsfield units (HUs)], low-density muscle (0–34 HU), total muscle (normal-density muscle plus low-density muscle), subcutaneous, and intermuscular fat CSA (cross-sectional area) by a technician in a blinded manner using SliceOmatic v4.2 software (Montreal, Canada) as previously described (32). All body composition measurements were conducted after an 8 h fast before and after the 6-mo training intervention within 72 h of the last exercise session.

### Physical Function

Stair-climb assessment was determined by participants ascending a 10-rise set of stairs as fast as possible, without holding on to a rail or using assistive devices. Repeated chair-rise time (10×) was determined on a standard chair with the participant holding their arms across the chest. Leg press strength and power were evaluated using pneumatic bilateral seated leg press equipment (K400, Keiser Sports Health Equipment Inc., Fresno, CA). Strength was defined as the 1-RM for the leg press. Peak power was also assessed for leg press. Following a baseline 1-RM measurement, each participant performed five repetitions, as fast as possible, at 40% and 70% 1-RM. The highest power output achieved during each of these five repetitions by a participant was designated as their peak power. All physical function tests were assessed before and after the 6-mo training intervention.

### Dietary Intake

Participants recorded food intake using 3-day food records at baseline and *month 6* of the study. Dietary intake data were analyzed using the Nutrition Data System for Research software version 2007 (Nutrition Coordinating Center, University of Minnesota, Minneapolis, MN). Energy and macronutrient intake reported as dietary intake plus whey protein or control beverage.

### Circulating miRNA Extraction and Expression

Circulating miRNA was extracted from 200 µL of serum using a miRNeasy serum plasma kit (Cat. No. 217184, Qiagen, Valencia, CA). Before RNA extraction, serum samples were centrifuged for 10 min at 4°C to remove potential contaminating cellular debris. A spike-in control [3.5 µL *C. elegans* (cel) miR-39; Cat. No. 219610, Qiagen] was added to all samples before RNA extraction to determine the yield of the template recovered.

A targeted approach was used for miRNA of interest (miR-1-3p, miR-19b-3p, miR-92a, miR-99b, miR-100-5p, miR-103, miR-126, miR-133a-3p, miR-133b, miR-206, miR-221, miR-320a, miR-378, miR-423, miR-486, miR-574, and miR-885) using TaqMan miRNA assays (Cat. No. 4427975, Thermo Fisher Scientific, Waltham, MA) and a multiplex RT and preamplification protocol (33). Targets of interest were chosen based on previous work from our laboratory (21, 25, 34) and others (23, 24, 35) showing aging and exercise altering specific miRNA. Extracted RNA was reverse-transcribed using the Taq-Man miRNA RT kit (Cat. No. 4366596, Thermo Fisher Scientific) with the miRNA-specific stem-loop RT primers pooled in 1X Tris-EDTA (TE) buffer for a final dilution of 0.05× for each miRNA RT primer. The RT primer pool (6 µL) was added to the RT reaction mix (0.3 µL of 100 mM dNTP, 3 µL of enzyme, 1.5 µL of 10× RT buffer, and 0.19 µL of RNase inhibitor) and 3 µL of serum RNA. A preamplification step was performed to increase the cDNA template using a primer pool of 20× TaqMan small RNA assay for the miRNA of interest at 0.05× concentration in 0.1× TE buffer. Preamplification reaction mix consisted of 3.75 µL of primer pool, 2.5 µL of cDNA, 12.5 µL of TaqMan Universal PCR master mix (2×) without UNG (Cat. No. 4440040, Thermo Fisher Scientific), and 6.25 µL of nuclease-free H_2_O. RT and preamplification were conducted using a thermal cycler (model T100, Bio-Rad, Hercules, CA). RT-qPCR amplifications were conducted using a real-time PCR detection system (CFX96 Touch, Bio-Rad). After preamplification, a sufficient template was present to quantify cDNA using a spectrophotometer (model ND-1000, Nanodrop Technologies, Wilmington, DE) to ensure that equal amounts were loaded per sample.

All miRNA were normalized using the geometric mean of spike-in control cel-miR-39 (external control) and RNU6 (internal control). Combination of external and internal controls allows for normalization to account for both technical and inter-individual variation (36). The geometric mean of spike-in control cel-miR-39 and RNU6 was determined to be a homogenously and stably expressed housekeeper with cycle thresholds (C_T_) being not different between groups or time (Loser PRE: 26.66 ± 0.19, POST: 26.21 ± 0.26; Gainers PRE: 26.54 ± 0.23, POST: 26.67 ± 0.21) and an overall coefficient of variation of 3.9% (37). Fold changes were calculated using the ΔΔC_T_ method, with average PRE data for Gainers and Losers set as control to calculate miRNA expression at PRE and POST (38).

### Bioinformatics Analysis

Following the initial analysis, miRNAs that were statistically different between groups (miR-1-3p, miR-19b-3p, miR-92a, miR-126, miR-133a-3p, and miR-133b) were uploaded to DNA Intelligent Analysis (DIANA) miRPath 3.0 (Alexander Fleming Biological Sciences Research Center, Athens, Greece; http://diana.imis.athena-innovations.gr) to determine potential Kyoto Encyclopedia of Genes and Genomes (KEGG; http://www.genome.jp/kegg/) pathways regulated by these miRNA using experimentally verified targets from TarBase 7.0 and microT-CDS (Alexander Fleming BSRC) (39).

### Animal Experiments

Male C57BL/6 mice aged 3 mo (6 mo, *n* = 6) and 21 mo (24 MO, *n* = 6) were purchased from the National Institute on Aging Aged Rodent Colony/Jackson Laboratory (Bar Harbor, ME). Mice were housed in a temperature-controlled animal room (21°C) maintained on a 12-h light-dark cycle with free access to food and water for the study duration (3 mo). All animal experimentation procedures were approved by the Institutional Animal Use and Care Committee of Tufts University.

#### Grip strength, body composition, and muscle mass.

A Chatillon E-DFE digital force gauge (Ametek, Largo, FL) was used to measure grip strength. Briefly, the forelimb of the mouse is allowed to grasp onto the pull bar assembly. The animal is then gently pulled backward by the tail leading away from the sensor until the grip is released and the maximum force attained and stored on the display. Three trials are performed for each mouse with a 10-min resting period between trials. Determination of the body composition of each animal was performed using quantitative magnetic resonance EchoMRI-900 whole body composition analyzer (Echo Medical Systems; Houston, TX). Plantaris muscle was extracted, the wet weight of the muscle was obtained, immediately snap frozen in liquid N_2_, and stored at −80°C until further miRNA analysis.

#### Circulating exosome isolation.

After a 5-h fast, ∼1 mL of blood was obtained from the jugular vein, allowed to clot for 30 min at room temperature (RT), centrifuged and the supernatant was collected as serum. Total exosomes were extracted from 500 µL of serum using miRCURY Exosome Serum/Plasma kit (QIAGEN) according to the manufacturer’s instructions. In brief, an initial spin was performed at 10,000 *g* (RT) for 10 min for each sample to remove cells and debris. The precipitation reagent was added to the sample volume, according to the manufacturer’s instructions. Mixtures were vortexed and incubated at 4°C for 1 h and then centrifuged at room temperature for 30 min at 1,500 *g* followed by pellet resuspension in resuspension buffer. All exosomes were stored at −80°C immediately after isolation until further analysis.

### In Vitro Analysis

#### Cell culture.

In vitro experiments were then conducted to confirm that miR-19b-3p indeed targets and alters pathways regulating skeletal muscle mass. HMCL-7304 human myotubes were derived from an immortalized myoblast cell line that had been established from the intercostal skeletal muscle of a female donor with no neuromuscular disorder (40) and obtained from MRC CNMD Biobank London (UCL Institute of Neurology, UK). Cells were cultured in a 1:1 ratio of PromoCell skeletal muscle cell growth medium (GM, PromoCell, Heidelberg, Germany) and DMEM/F12 supplemented with 20% FBS (Atlanta Biologicals, GA) and 1% antibiotic/antimycotic (ABAM, Gibco/Thermo Fisher Scientific). Differentiation to myotubes was induced by culture in PromoCell skeletal muscle differentiation medium (DM, PromoCell) and, after 5 days, multinucleated myotubes were visible under low magnification.

#### Cell transfection.

All transfections were performed at a minimum in triplicate. Cells were transfected overnight for up to 16 h, using either hsa-miR-19b-3p miRCURY LNA miRNA Mimic (YM00470545-ADB, Exiqon/Qiagen) or negative control miRCURY LNA miRNA Mimic (YM00479902-ADB, Qiagen). The 5′ ends of both nucleotides were marked with 6-carboxyfluorescein (*6-FAM*). Equal volumes of RNAiMax lipofectamine (Thermo Fisher Scientific) were used for a final concentration of 40 nM. Transfections were performed on cells that were differentiated for a minimum of 5 days.

#### In vitro anabolic capacity.

Protein synthesis was measured by the SUrface SEnsing of Translation (SUnSET) technique via immunological detection of puromycin labeled peptides as described previously (41). Briefly, HMCL-7304 cells were serum-starved for 2 h. Then IGF-1 (100 ng/mL, long-R3-IGF-1, MilliporeSigma) or IGF-1 and rapamycin (RAPA; 150 ng/mL, R8781, MilliporeSigma) or vehicle (VEH) were added to the cell culture media for 2 h. Subsequently, puromycin (1 µM, MilliporeSigma) was added 30 min before harvest.

#### Western blotting.

The phosphorylation and concentration of signaling proteins were quantified via Western blotting as described previously (41). After the experiments, the cells were collected in RIPA Lysis and Extraction Buffer (Thermo Fisher Scientific) supplemented with cOmplete ULTRA (Roche Applied Science, Indianapolis, IN) and PhosSTOP inhibitors (1 tablet/10 mL; Roche). Following centrifugation (10,000 *g* at 4°C) for 10 min, the supernatant was collected and assayed for protein content. The supernatant was solubilized in Laemmli buffer (10 mM DTT), separated by SDS-PAGE, and transferred to PVDF membranes. The membranes were then blocked (5% nonfat dry milk) and incubated overnight at 4°C with primary antibodies specific for phosphatase and tensin homolog deleted on chromosome 10 (PTEN; Cat. No. 9559; AB_390810), phosphorylated ribosomal protein S6 (phospho-rpS6; Cat. No. 2215; AB_331682), rpS6 (Cat. No. 2217; AB_331355), phospho-p70 S6 kinase (S6K1; Cat. No. 9205; AB_330944) and S6K1 (Cat. No. 2880; AB_390722), phospho-Akt (Cat No. 4060; AB_2315049) and Akt (Cat. No. 4685; AB_2225340), phospho-Forkhead box protein O1 (FOXO1; Cat. No. 84192; AB_2800035), and FOXO1 (Cat. No. 2880; AB_2106495). Membranes were probed with GAPDH (Cat. No. 2118; AB_561053) to monitor protein loading; all antibodies were from Cell Signaling Technology (Danvers, MA), and antipuromycin (MABE343; AB_2566826) was from MilliporeSigma (Burlington, MA). All primary antibodies were diluted at 1:1,000. Secondary antibody (anti‐rabbit IgG conjugate with horseradish peroxidase; Cell Signaling Technology) was applied to label primary antibodies at a 1:2,000 dilution, except GAPDH which was a 1:10,000 dilution. The immunoreactive proteins were detected with SuperSignal Chemiluminescent Substrate (Thermo Fisher Scientific, Rockford, IL), and intensities were quantified by densitometry (Bio-Rad ChemiDoc XRS+ system; San Leandro, CA) and analyzed as previously described (42).

#### In vitro/in vivo quantitative PCR analysis.

Total RNA was extracted using the miRNeasy Tissue/Cells Advanced Mini kit per manufacturer’s instructions (217604, Qiagen). RNA concentration and purity were determined by spectrophotometry (Nanodrop 1000, Thermo Fisher Scientific, Wilmington, DE). cDNA for mRNA analysis was generated via iScript Reverse Transcription Supermix for RT-qPCR (170–8840, Bio-Rad). cDNA were measured using PrimePCR SYBR Green Assay **(**BioRad) for the following genes: PTEN (Unique Assay ID: qHsaCED0036796), DROSHA (Unique Assay ID: qHsaCID0014729), DICER1 (Unique Assay ID: qHsaCID0006710), NRF1 (Unique Assay ID: qHsaCID0007351), TFAM (Unique Assay ID: qHsaCED0037846), PRKAA2 (Unique Assay ID: qHsaCED0045979), PPARGC1A (Unique Assay ID: qHsaCID0006418), FBXO32 (Unique Assay ID: qHsaCID0036673), TRIM63 (Unique Assay ID: qHsaCID0011239). Fold change in gene expression was normalized to the reference gene: GAPDH (Unique Assay ID: qHsaCEP0041396**)**. cDNA for miRNA analysis was generated via miRCURY LNA RT kit (339340, Qiagen). cDNA was measured using miRCURY LNA miRNA PCR Assay (Exiqon, Qiagen) for the following targets: hsa-miR-19b-3p (Cat. No. YP00204450, PRO No. 339306) and reference genes: U6 snRNA (Myocytes; Cat. No. YP00203907, PRO No. 339306) or SNORD65 (Exosomes; Cat. No. YP00203910, PRO No. 339306). All reactions were run using a commercially available reaction mixture for mRNA targets (iTaq Universal SYBR Green Supermix, Bio-Rad) and miRNA targets (miRCURY LNA SYBR Green PCR Kit, Qiagen) on a CFX-96 Touch (Bio-Rad).

### Statistical Analysis

Baseline between group (Losers vs. Gainers) differences for age, total body mass, fat mass, fat-free mass, and energy and macronutrient intake were determined using independent *t* tests. Chi-square analysis was used to determine differences in sex (men vs. women) and supplement (whey protein vs. placebo) between Losers and Gainers. Independent *t* test was used to assess differences between Losers in Gainers for delta total body mass, fat mass, fat-free mass, and physical performance outcome measurements.

Circulating miRNA data were not normally distributed (*P* < 0.05) as determined by Shapiro–Wilk tests. Fold change data for circulating miRNA were log-transformed (log_2_) for statistical analysis. Mixed model repeated measures ANOVA was used to assess the effect of group (Losers vs. Gainers) and time (PRE vs. POST) and their interactions for circulating miRNA expressions. Unstructured was determined as the appropriate covariance model based on the Akaike’s information criterion. Bonferroni corrections were used for pairwise comparisons of significant interactions. Spearman’s rank correlation coefficient was used to determine the correlation of circulating miRNA to changes in fat-free mass and fat mass for Losers and Gainers. Significance was set at *P* < 0.05. Data were analyzed using IBM SPSS Statistics for Windows v. 22.0 (IBM Corp., Armonk, NY).

In the in vitro experiments, differences between groups were identified using either a *t* test or a two-way ANOVA with Bonferroni post hoc test with GraphPad Prism 5.00 for Windows. Results are expressed as means ±SD, and statistical significance was accepted at *P* < 0.05.

Differences between groups in the animal experiments were identified using a *t* test with GraphPad Prism 5.00 for Windows. Skeletal muscle miR-19b-3p data was not normally distributed (*P* < 0.05) as determined by the Shapiro-Wilk test. Fold change data for skeletal muscle miR-19b were log-transformed (log_2_) for statistical analysis. Results are expressed as means ± SE, and statistical significance was accepted at *P* < 0.05. Spearman’s rank correlation coefficient was used to determine the correlation of skeletal muscle miR-19b-3p to changes in fat-free mass, fat mass, and grip strength for younger (6 mo) and older (24 mo) animals.

## RESULTS

### Body Composition and Physical Function

Chi-square analysis confirmed no difference in the number of men and women or participants consuming the whey protein and placebo supplement dichotomized into Losers or Gainers (Table 1). All participants experienced a change in body mass following the 6-mo training protocol (Table 3). Total body mass and fat-free mass increased (*P* < 0.001) from PRE to POST in Gainers and this change was greater (*P* < 0.001) compared with Losers (Fig. 1*A*). Fat mass decreased (*P* = 0.008) from PRE to POST in Loser and this change was greater (*P* = 0.01) compared with Gainers (Fig. 1*A*). Fat-free mass did not change from PRE to POST in Losers (Table 3). Both groups increased (*P* = 0.02) leg cross-sectional area with Gainers demonstrating a numerical higher, but not statistically different (*P* = 0.16), increases in the cross-sectional area compared with Losers (Table 3).

**Table 3.** Body composition and leg cross-sectional area

	Losers, *n* = 33		Gainers, *n* = 40			*P* value	
	PRE	POST	PRE	POST	Time	Group	Time-by-Group
Total body mass, kg	75.8 ± 10.3	74.8 ± 10.4†	73.4 ± 9.8	74.7 ± 10.8†	0.55	0.59	<0.001
Fat mass, kg	26.6 ± 6.3	25.9 ± 7.6†	25.0 ± 6.9	25.2 ± 7.0	0.16	0.50	0.01
Fat-free mass, kg	47.0 ± 8.6	46.8 ± 9.5	46.2 ± 7.5	47.3 ± 8.2*†	0.01	0.93	<0.001
Cross-sectional area	97.3 ± 23.6	98.5 ± 28.5†	97.4 ± 20.68	102.1 ± 24.7†	0.02	0.75	0.16

Values means ± SE. No differences in baseline characteristics between losers and gainers.

*Different than loser; *P* < 0.05.

†Different than pre; *P* < 0.05. Statistical analysis mixed model repeated measures ANOVA with Bonferroni correction.

Physical function assessment for chair raise times improved to a greater (*P* = 0.04) extent in Gainers compared with Losers. Increases in 1-RM leg press and peak leg power at 40% of maximal effort were higher (*P* < 0.05) in Gainers compared with Losers (Fig. 1*B*). No other differences were observed between Gainers and Losers in physical function outcomes reported in the parent study (32).

Physical function assessment for stair climb and chair raise times improved to a greater (*P* < 0.05) extent in Gainers compared with Losers (Fig. 1*B*). Increases in 1-RM leg press and peak leg power at 40% and 70% of maximal effort were higher (*P* < 0.05) in Gainers compared with Losers (Fig. 1*B*). No other differences were observed between Gainers and Losers in physical function outcomes reported in the parent study (32).

### Circulating microRNA

**Figure 2.**
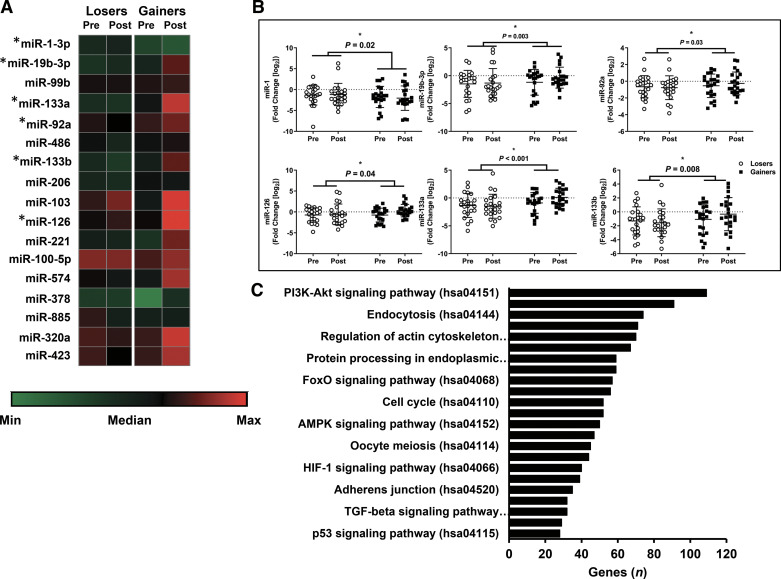
Heatmap of targeted measurement of circulating miRNA (*A*), individual miRNA that were different between Losers and Gainers (*B*), bioinformatic identification of significant (*P* < 0.05) and relevant KEGG pathways associated with anabolic regulation by the measured miRNA (*C*). Data represent means ± SD (Losers; *n* = 33 and Gainers; *n* = 40). Dots are individual data points. *Different than Losers; *P* < 0.05. Statistical analysis mixed model repeated measures ANOVA with Bonferroni correction.

**Figure 3.**
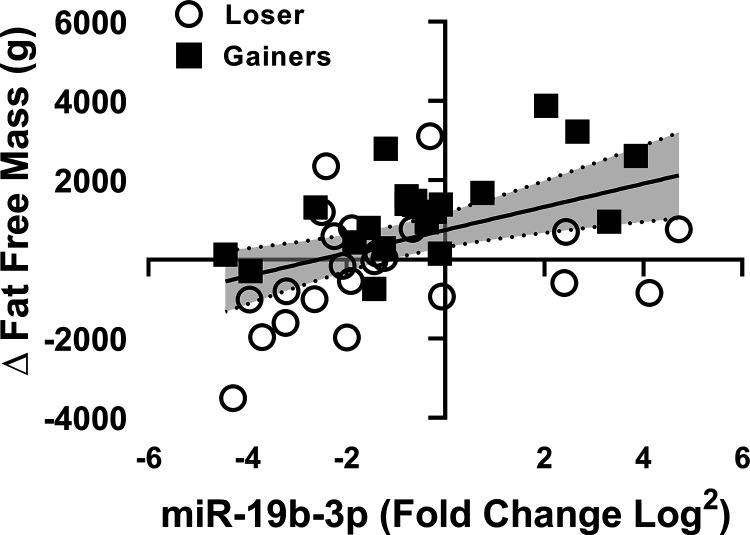
Associations of miR-19b-3p and fat-free mass in Losers and Gainers (Losers; *n* = 33 and Gainers; *n* = 40).

Of the 17 miRNA (Fig. 2*A*) assessed in this analysis, 5 miRNA (miR-19b-3p, miR-92a, miR-126, miR-133a-3p, and miR-133b) were higher (*P* < 0.05) and 1 miRNA (miR-1-3p) was lower (*P* < 0.05) in Gainers compared with Losers (Fig. 2*B*). Assessment of pathways targeted by all 6 miRNA identified that the PI3K-Akt signaling pathway had the most genes targeted (Fig. 2*C*). Other pathways regulating muscle mass such as MAPK, FOXO, and mTOR signaling, and ubiquitin mediated proteolysis were also identified by bioinformatics analysis. Of the six miRNA that were different between Gainers and Losers, miR-19b-3p was the most highly associated with change in whole body fat-free mass (*r* = 0.490, *r*^2^ = 0.202, *P* < 0.05; Fig. 3), with the strongest association observed in Gainers (*r* = 0.615, *r*^2^ = 0.383, *P* < 0.05) compared with Losers (*r* = 0.409, *r*^2^ = 0.080, *P* = 0.06).

### miR-19b-3p Expression is Affected by Aging and Associated with Higher Lean Mass and Strength in Mice

**Figure 4.**
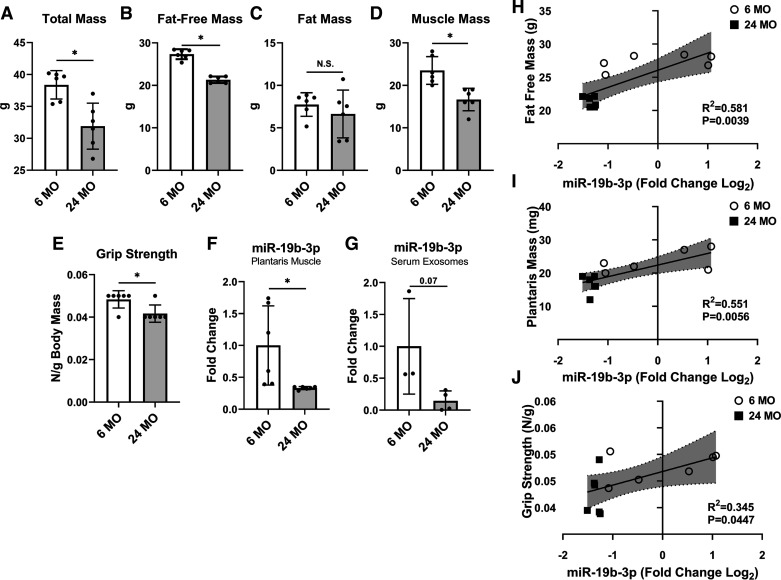
Difference in body mass (*A*), fat-free mass (*B*), and fat mass (*C*) by quantitative magnetic resonance, muscle mass (*D*), and grip strength (*E*) in young (6 mo) and older (24 mo) mice. miR-19b-3p expression in skeletal muscle (*F*) and circulation (*G*). Associations of miR-19b-3p and fat-free mass (*H*), muscle mass (*I*), and strength (*J*) in younger and older mice. Data represent means ± SD (*n* = 3–6). Dots are individual data points *Different than 6 mo; *P* < 0.05. Statistical analysis unpaired *t* test.

Similar to humans, muscle mass is lost with aging in mice, as reported in studies comparing mice aged 24-mo to mice 15-mo old and younger (41, 43, 44). In the current study, we find that 6 mo had ∼20% higher body mass (Fig. 4*A*) and fat-free mass (Fig. 4*B*), but no significant differences in fat mass (Fig. 4*C*) compared with 24 mo mice. Plantaris muscle mass (Fig. 4*D*), a muscle with a higher proportion of white fibers, was ∼40% higher in 6 mo versus 24 mo. Six month had 12% greater grip strength (Fig. 4*E*) compared with 24 mo. The expression of miR-19b-3p in skeletal muscle (Fig. 4*F*) was 5× higher in 6 mo compared with 24 mo, whereas miR-19b-3p expression trended higher (*P* = 0.07) in the serum exosomes (Fig. 4*G*) of 6 mo versus 24 mo. miR-19b-3p was positively associated with whole body fat-free mass (*r*^2^ = 0.581, *P* = 0.0039; Fig. 4*H*), plantaris muscle mass (*r*^2^ = 0.551, *P* = 0.0056; Fig. 4*I*), and grip strength (*r*^2^ = 0.345, *P* = 0.0447; Fig. 4*J*). These data provide evidence that miR-19b-3p expression in skeletal muscle is associated with aging-induced muscle atrophy and decreased strength in mice.

### In Vitro Analysis

Given the potential for these circulating miRNA to target pathways regulating muscle mass, and that miR-19b-3p was most highly associated with a change in fat-free mass, additional cell culture experiments were carried out specifically targeting the influence of miR-19b-3p on regulating muscle anabolism and catabolism.

### Overexpression of miR-19b-3p Decreases PTEN Expression in Human Myocytes

miR-19b-3p is a member of the oncogenic miRNA cluster, the miR-17–92 cluster, and has been shown to play an essential role in cell proliferation and has anti-apoptosis effects, and along with other members of the miR-17–92 cluster were identified as a novel biomarker of cellular aging (45–48). Phosphatase and tensin homolog (PTEN) is a tumor suppresser gene that has been identified as a target of miR-19b that is responsible for its anti-apoptosis effects (45–48). Therefore, we wanted to determine if miR-19b-3p overexpression in vitro in human skeletal muscle induces similar regulation of PTEN that was previously determined in silico and the expression of the RNase III nucleases Drosha and Dicer, critical mediators of global miRNA biosynthesis.

**Figure 5.**
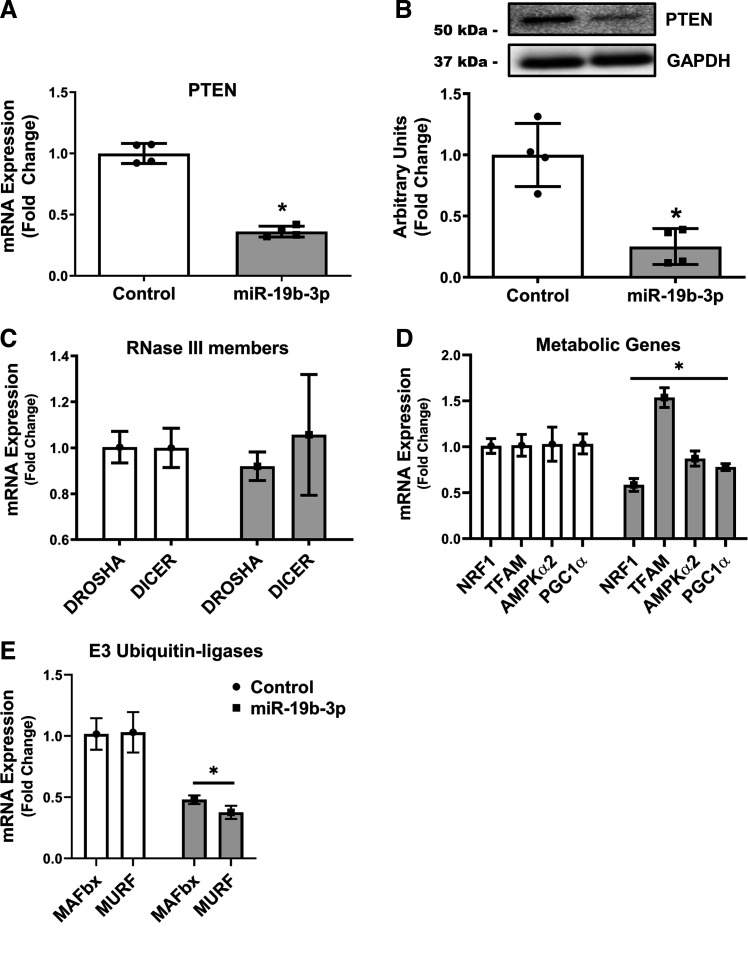
Human myocytes (HMCL-7304) were transfected with negative control (Control) or mimic (miR-19b-3p) for 48 h. mRNA (*A*) and protein expression (*B*) of the miR-19b-3p target, PTEN, RNAse III members (*C*), metabolic genes (*D*), and muscle specific E3 ubiquitin-ligases (*E*) that regulate skeletal muscle health were then measured. Data represents means ± SD (*n* = 3). *P < 0.05 vs. Control by *t* test. PTEN, phosphatase and tensin homolog.

We observed that overexpressing miR-19b-3p led to a 64% significant decrease in the mRNA expression of its predicted target PTEN (Fig. 5*A*). Using Western blotting analysis, we measured the protein expression of PTEN in human myocytes. Similar to the mRNA results, PTEN was significantly decreased in these cells by 75% (Fig. 5*B*) compared with the scramble cells. Alternatively, the expression of DICER and DROSHA (Fig. 5*C*), remained unchanged, suggesting a lack of impact of miR-19b-3p on miRNA biogenesis.

### miR-19b Overexpression Had Varied Effects on Regulators of Energy Homeostasis in Skeletal Muscle

miR-19 has previously been associated with targeting proteins implicated in cellular growth (49) and mitochondrial dysfunction and energy substrate metabolism mediating the divergent metabolic health profiles of rats bred for low (LCR) and high (HCR) capacity running (50–52). We determined if mitochondrial genes and/or genes that regulate oxidative metabolism are affected by miR-19b-3p overexpression in human myocytes. Therefore, we measured the gene expression of the nuclear transcription factors peroxisome proliferator-activated receptorγ coactivator-1 (PGC-1α), mitochondrial transcription factor A (TFAM), nuclear respiratory factor 1 (NRF1), and the cellular energy regulator AMP-activated protein kinase (AMPK)α2. miR-19b-3p overexpression induced declines in both NRF1 and PGC-1α expression in concert with significant increases in TFAM expression (Fig. 5*D*). We next measured atrogin-1 (MAFbx) and MuRF1, the two major muscle-specific ubiquitin E3 ligases, and found that both were significantly decreased ∼55% with miR-19b-3p overexpression (Fig. 5*E*). These diverse changes in the expression of key regulators of cellular growth and energy homeostasis suggest miR-19b-3p may play a regulatory role in skeletal muscle metabolism.

### miR-19b-3p Regulates Skeletal Muscle Growth and Anabolic Capacity in Human Myocytes

**Figure 6.**
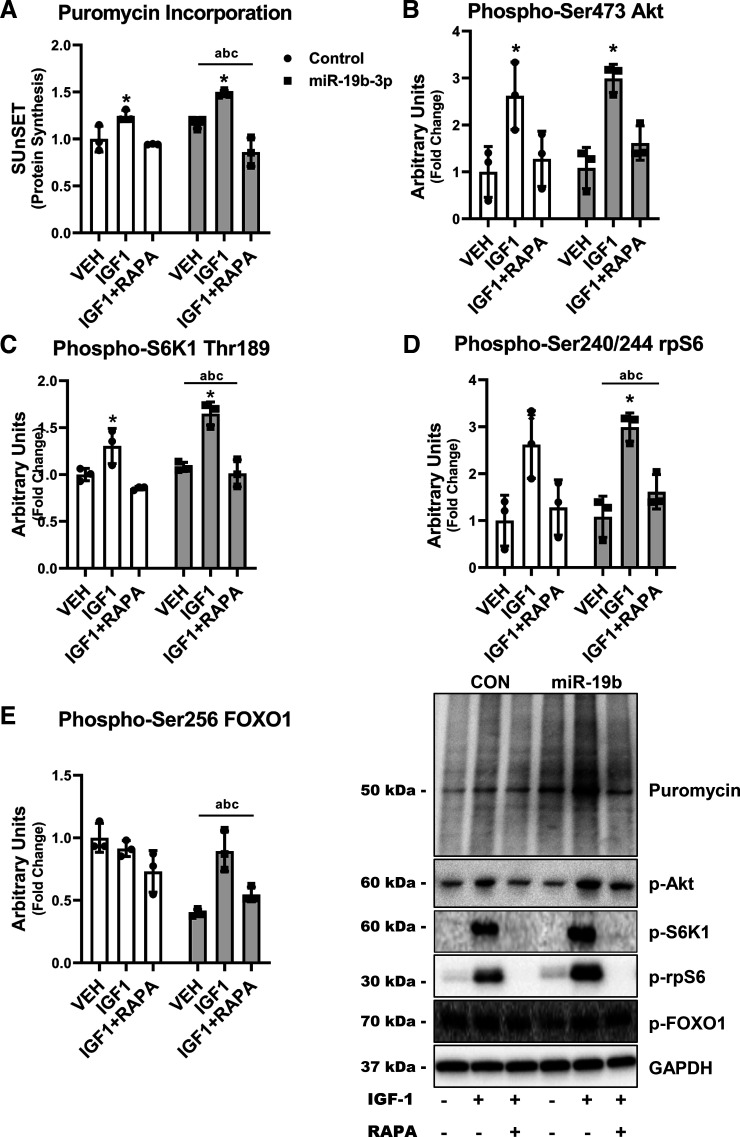
After 48 h of transfection of negative control (Control) or mimic (miR-19b-3p), human myocytes were treated with (IGF1) or without (VEH) 100 ng/mL IGF-1 or IGF-1 and 150 ng/mL rapamycin (IGF1+RAPA). Protein synthesis was determined using the SUnSET assay (*A*). The activation of anabolic signaling was determined by the phosphorylation of Akt (*B*), S6K1 (*C*), rpS6 (*D*), and FOXO1 (*E*) after IGF-1 with or without rapamycin. Data represent means ± SD (*n* = 3). ^a^*P* < 0.05 for main effect phenotype, ^b^*P* < 0.05 for main effect treatment, ^c^interaction phenotype-treatment, *P < 0.05 vs. vehicle (VEH). FOXO1, forkhead box protein O1.

The loss of PTEN in skeletal muscle has been previously reported to inducing muscle hypertrophy (53, 54). Therefore, we wanted to determine if decreased PTEN expression via the overexpression of miR-19b-3p would increase the anabolic response to IGF-1 in human myocytes. Protein synthesis was significantly increased ∼50% after IGF-1 in miR-19b-3p overexpressed myocytes compared with only ∼30% in Control even though overexpression could not prevent atrophic effects of rapamycin in these cells (Fig. 6*A*). We next determined the IGF-1 activation of anabolic signaling in human myocytes. Mirroring the protein synthesis results, IGF-1 significantly increased the phosphorylation of Akt (Fig. 6*B*) 2.6-fold in Control compared with 3-fold in miR-19b-3p overexpressed and S6K1 30% and 65% in Control and miR-19b-3p overexpressed myocytes, respectively (Fig. 6*C*) and the phosphorylation of rpS6 3.5-fold in miR-19b-3p overexpression compared with twofold in Control myocytes (Fig. 6*D*). In contrast, the phosphorylation of FOXO1 was decreased ∼60% with miR-19b-3p overexpression compared with Control while IGF-1 treatment reversed this effect (Fig. 6*E*). These data show that the anabolic capacity of human myocytes is highly regulated by miR-19b-3p expression in skeletal muscle.

## DISCUSSION

In this secondary analysis of mobility-limited older adults that underwent a 6-mo progressive resistance exercise training (PRET) intervention and randomized to receive either 40 g/day of a whey protein concentrate or an isocaloric control, we dichotomized participants by the gain (Gainers; *n* = 40) or loss (Losers; *n* = 33) of leg lean mass after PRET. The Losers experienced declines in body mass due to loss of fat mass. In contrast, Gainers increased body mass, due to a significant increase in fat-free mass. Significantly, gains in fat-free mass were associated with considerably more significant improvements of functional outcomes such as timed stair climb and chair-rise, one-repetition maximum strength, and peak power output in Gainers after PRET compared with Losers. To determine if any of the miRNA we have previously identified in an unbiased screen (21, 25) may predict gains in fat-free mass with PRET, we measured their circulating levels before and after training in a subset of participants (*n* = 22 Gainers; *n* = 22 Losers). We identified higher expression of circulating miR-19b-3p in Gainers versus Losers and the miRNA was most highly associated with fat-free mass gains following PRET. We next confirmed a relationship between miR-19b-3p expression in whole muscle with muscle mass and strength in older and younger mice. To determine a mechanistic role of miR-19b-3p on the regulation of muscle anabolism, we overexpressed miR-19b-3p in human myotubes. We found that miR-19b-3p overexpression reduced PTEN gene and total protein expression, augmented anabolic signaling, and caused an increase in cell growth and muscle protein synthesis at baseline and in response to IGF-1 stimulation. Our data from humans and a preclinical model of aging mice and human myotubes identify miR-19b-3p as a circulating biomarker of fat-free mass gains in older adults in response to PRET and support a role for miR-19b-3p as a positive regulator of skeletal muscle mass and anabolism.

To determine potential therapeutic targets for the prevention and treatment of sarcopenia, there is a need to better understand the underlying molecular processes regulating skeletal muscle anabolism. Similarly, understanding the molecular perspectives of anabolic resistance and/or the nonresponder phenomena stands out as notable hurdles to the development of effective interventions to combat sarcopenia. Our group has previously identified the dysregulation of miRNA in circulation (25) and skeletal muscle (21), with aging as a central mechanism resulting in muscle mass declines. Findings from the current investigation expand upon this work, reporting that circulating miR-19b-3p expression was higher in Gainers than in Losers. A higher expression of miR-19b-3p was associated with more significant gains in FFM following 6 mo of PRET. These results are consistent with our previous work identifying miR-19b-3p as a predictive marker for aging and “anabolic resistance” (25). Specifically, circulating miR-19b-3p was more highly expressed in younger than older adults under resting fasted conditions and positively associated with FFM (25). Furthermore, following a single bout of resistance exercise, principal component analysis identified the expression of miR-19b-3p, in addition to multiple members of the miRNA17 ∼ 92 cluster, was higher in younger compared with older adults (25). Increases in extracellular and cardiac miR-19b-3p, alongside other miR-17 ∼ 92 cluster members, have demonstrated a role in postexercise cardiac hypertrophy (55–57). In contrast, their downregulation has been observed in muscle myopathies such as muscular dystrophy and diabetic myopathy (58, 59). In addition to these prior findings, our results provide evidence of a role for miR-19b-3p in the anabolic response of skeletal muscle to exercise and a possible role in anabolic resistance found in aging.

Differences in circulating (60) and skeletal muscle (61) miRNA profiles have previously been used to assess anabolic heterogeneity following exercise training in younger adults. However, to the best of our knowledge, this is the first investigation utilizing differences in circulating miRNA expression profiles to explain the variance in older individuals’ training responses, more specifically older adults with mobility-limitations. Establishing predictive markers to determine inter-individual response to exercise is important as this may aid in structuring appropriate exercise or nutritional regimens to maximize the health benefits of training. This is particularly important for an aging population to ensure training adaptations result in desired muscle mass and function improvements. In the current study, we observed a high variability degree in response to PRET between Gainers and Losers. After training, Losers experienced declines in body mass, primarily from fat mass, whereas Gainers increased body mass due to increased fat-free mass. Compared with Losers, the gains of fat-free mass in the Gainers were associated with superior functional outcomes such as timed stair climb and chair-rise, one-repetition maximum strength, and peak power output after PRET. Our results provide evidence not only for the variability of exercise-response in older adults but a further diametric response in these individuals. Results from the current study expand on our previous findings, indicating miR-19b-3p is not only a predictive marker for aging but also a marker of PRET responses within an older frail population. Higher expression of circulating miR-19b-3p in younger adults and Gainers suggests this miRNA may have a functional role in maintaining or increased muscle mass. A limitation of the current study is the inability to definitively determine the tissue-specific origin of miR-19b-3p in the circulation of the Gainers and Losers. A large majority of miRNA found in circulation are endogenously transcribed in cells not produced in the circulation (62). Given this evidence any changes of expression tissue-specifically would likely be mirrored in circulation. Previous studies have reported a clear link with changes in miRNA biogenesis in tissue and the levels of circulating miRNAs in humans and mice (63–65). However, more mechanistic experiments were required to elucidate the effect of higher circulating miR-19b-3p on skeletal muscle mass’s molecular regulation.

There is strong evidence suggesting extracellular miRNA in circulation is more than just byproducts of cellular activity but function in cell-cell signaling during various physiological and pathological processes (66–68). The miRNA17–92 cluster is a polycistronic miRNA that produces six individual miRNAs (miR-17, miR-18a, miR-19a, miR-19b, miR-20a, and miR-92a), governing various biological functions that regulate multiple cellular processes involved in cell proliferation, differentiation, proteolysis, and survival (49, 69–73). miR-19b-3p is a well-described member of the miRNA17–92 cluster family and, along with other members was identified as a novel biomarker of cellular aging (71). In human skeletal muscle cells, we overexpressed miR-19b-3p and determined the expression of a selection of its predicted targets and the response of these cells to anabolic stimulation. We have now determined that miR-19b-3p regulates skeletal muscle protein synthesis through its inhibition of PTEN. PTEN’s role as a phosphatase and tumor suppressor has recently gained attention as a metabolic regulator and important growth and survival regulatory gene (74, 75). Emerging studies have identified additional roles for PTEN in muscle hypertrophy, regeneration, and satellite cell maintenance (54, 75, 76). Here we show the overexpression of miR-19b-3p in human skeletal muscle cells decreased the expression of PTEN mRNA transcript and protein levels in these cells. The decrease of PTEN was associated with increased protein synthesis after IGF-1 stimulation in these muscle cells. Importantly, this is in line with PTEN’s demonstrated role as a protein phosphatase preventing the activation of PIP3 binding effector proteins such as AKT (77). These results reflect our previous work in humans that revealed circulating miR-19b-3p expression was associated with skeletal muscle phosphorylation status of the anabolic signaling proteins AKT and S6K1, suggesting lower expression of miR-19b-3p in older adults was predictive of “anabolic resistance” (25). However, the overexpression of miR-19b-3p in skeletal muscle cells was unable to overcome rapamycin treatment during IGF-1 stimulation. These results confirm previous work revealing that rapamycin treatment effectively rescued the established hypertrophic cardiomyopathy found in the cardiomyocyte-specific knockout of PTEN (78). This provides evidence that the actions of miR-19b-3p in human muscle cells were the result of the inhibition of PTEN that are upstream of the activities of mTOR.

The FoxO1/MuRF1/atrogin-1 ubiquitin/proteasome axis, of which its members are classified as “atrogenes,” is a pathway with multiple regulatory roles including, but not limited to oxidative stress, apoptotic cell death, catabolism and insulin sensitivity (75, 79–82). Here we report the overexpression of miR-19-3p decreased the phosphorylation of the FOXO1 transcription factor and its transcriptional targets atrogin-1 and MURF1. This is in line with a previous report that miR-19b can regulate cardiac hypertrophy and survival through the repression of the atrogin-1 and MuRF-1 (49). These results may provide evidence that PTEN and its subsequent effects on AKT inhibited FOXO translocation into the nucleus of the cells to act as a transcription factor for atrogin-1 and MURF1. This data suggest that miR-19b-3p not only improves anabolism but also reduces catabolism to further enhance protein balance to support gains in muscle mass.

Utilizing a preclinical mouse model of aging, we determined an in vivo role for miR-19b-3p expression in skeletal muscle and circulating exosomes on sarcopenia. In older animals, we observed both muscle atrophy and weakness compared with younger animals. The use of preclinical models such as, mice has been demonstrated to mirror the molecular changes observed in human sarcopenia (83). Here we observed that miR-19b-3p expression in the skeletal muscle was decreased with aging. This is similar to previous studies that have reported decreased miR-19b expression in human aging in multiple cell types (71, 84). In mice, we observed a higher expression of miR-19b-3p was associated with higher fat-free mass, muscle mass, and strength. These results correspond with our human data in circulation showing the relationship between miR-19b-3p expression and increased muscle mass and physical function.

In summary, we utilized the physiological heterogeneity of the anabolic response of mobility-limited older adults that undertook 6 mo of progressive resistance exercise training. Here we report two diametric responses to PRET in these elderly individuals with an equal number that either increased (Gainers) or decreased (Losers) leg-lean mass independent of nutritional status. The increases of leg-lean mass in Gainers were associated with considerably more significant enhancements in physical outcomes after PRET compared with Losers. Using targeted genomic and bioinformatics analysis of circulating miRNA profiles in these two distinct groups revealed that miR-19b-3p was associated with more significant FFM gains following 6 mo of PRET. This novel finding is of critical importance because of the recently reported role of extracellular miRNA in circulation on cell-cell communication during various physiological and pathological processes. In follow-up mechanistic work in human skeletal muscle cells, we found that the overexpression of miR-19b-3p inhibits the expression and translation of PTEN, which allowed for increased mTORC1 anabolic signaling and increased protein synthesis in these cells. Using integrative physiological experiments in humans, human skeletal muscle cells, and mice, we have discovered a role for miR-19b-3p in anabolic capacity and in the anabolic heterogeneity in mobility-limited older adults. Our results have the ability to further what is known about the variability to exercise response and provide the potential to investigate the possibility of miR-19b-3p and other miRNAs in circulation as a therapeutic target for the age-associated loss of skeletal muscle mass and function.

## GRANTS

This work was supported by National Institutes of Health (NIH)/National Institute on Aging (NIA) Grants K01-AG-047247 and 1-P30-AG-031679 (to D. A. Rivas), Dairy Research Institute (R. A. Fielding), The Sao Paulo Research Foundation FAPESP 2017/17646-1 (A. S. R. da Silva), the US Department of Agriculture under agreement No. 58–1950-4-003 (R.A. Fielding), and the US Army Medical Research and Development Command (L. M. Margolis).

## DISCLAIMERS

Any opinions, findings, conclusions, or recommendations expressed in this publication are those of the authors and do not necessarily reflect the view of the US Department of Agriculture (USDA), the US Department of the Army (DA) or US Department of Defense (DOD). Mention of trade names or commercial products in this publication is solely for the purpose of providing specific information and does not imply recommendation, nor constitute endorsement by the USDA, DA or DOD. The investigators adhered to the policies for protection of human subjects as prescribed in US Army Regulation 70-25, and the research was conducted in adherence with the provisions of 32 CFR part 219.

## DISCLOSURES

No conflicts of interest, financial or otherwise, are declared by the authors.

## AUTHOR CONTRIBUTIONS

D.A.R., R.A.F., and L.M.M. conceived and designed research; D.A.R., F.P., T.B., A.S.R.d.S., R.A.F., and L.M.M. performed experiments; D.A.R., F.P., and L.M.M. analyzed data; D.A.R., F.P., T.B., A.S.R.d.S., R.A.F., and L.M.M. interpreted results of experiments; D.A.R. and L.M.M. prepared figures; D.A.R., A.S.R.d.S., and L.M.M. drafted manuscript; D.A.R., F.P., T.B., A.S.R.d.S., R.A.F., and L.M.M. edited and revised manuscript; D.A.R., F.P., T.B., A.S.R.d.S., R.A.F., and L.M.M. approved final version of manuscript.
